# Clinical features and multiomics profiles indicate coagulation and platelet dysfunction in COVID-19 viral sepsis

**DOI:** 10.1016/j.isci.2024.110110

**Published:** 2024-05-25

**Authors:** Zhiqing Xiao, Minggui Lin, Ning Song, Xue Wu, Jingyu Hou, Lili Wang, XinLun Tian, Chunge An, Charles S. Dela Cruz, Lokesh Sharma, De Chang

**Affiliations:** 1 Department of Pulmonary and Critical Care Medicine at The Seventh Medical Center, College of Pulmonary and Critical Care Medicine of The Eighth Medical Center, Chinese PLA General Hospital, Beijing 100853, China; 2 Hebei North University, Zhangjiakou 075000, Hebei, China; 3 Beijing Tsinghua Changgung Hospital, Tsinghua University School of Medicine, Beijing 102218, China; 4 Department of Infectious Diseases, The Second Hospital of Hebei Medical University, Shijiazhuang, Hebei 050000, China; 5 Department of Pulmonary and Critical Care Medicine, Peking Union Medical College Hospital, Chinese Academy of Medical Sciences & Peking Union Medical College, Beijing, China; 6 Division of Pulmonary, Allergy, Critical Care, and Sleep Medicine, Department of Medicine, University of Pittsburgh School of Medicine, Pittsburgh, PA 15213, USA

**Keywords:** health sciences, immunology, virology, proteomics, metabolomics

## Abstract

Increased cases of sepsis during COVID-19 in the absence of known bacterial pathogens highlighted role of viruses as causative agents of sepsis. In this study, we investigated clinical, laboratory, proteomic, and metabolomic characteristics of viral sepsis patients (*n* = 45) and compared them to non-sepsis patients with COVID-19 (*n* = 186) to identify molecular mechanisms underlying the pathology of viral sepsis in COVID-19. We identified unique metabolomic and proteomic signatures that suggest a substantial perturbation in the coagulation, complement, and platelet activation pathways in viral sepsis. Our proteomic data indicated elevated coagulation pathway protein (fibrinogen), whereas a decrease in many of the complement proteins was observed. These alterations were associated with the functional consequences such as susceptibility to secondary bacterial infections and potentially contributing to both local and systemic disease phenotypes. Our data provide novel aspect of COVID-19 pathology that is centered around presence of sepsis phenotype in COVID-19.

## Highlights

• COVID-19 viral sepsis contributes to multiple organ injuries and immune impairment
• Viral sepsis increased susceptibility to secondary infections and increased mortality
• Dysregulation of the coagulant and complement pathways was involved in viral sepsis
• C4b, C8g, Lum, RTN4, and KNG-1 are potential biomarkers for COVID-19 virus sepsis

## Graphical abstract

**Figure:**
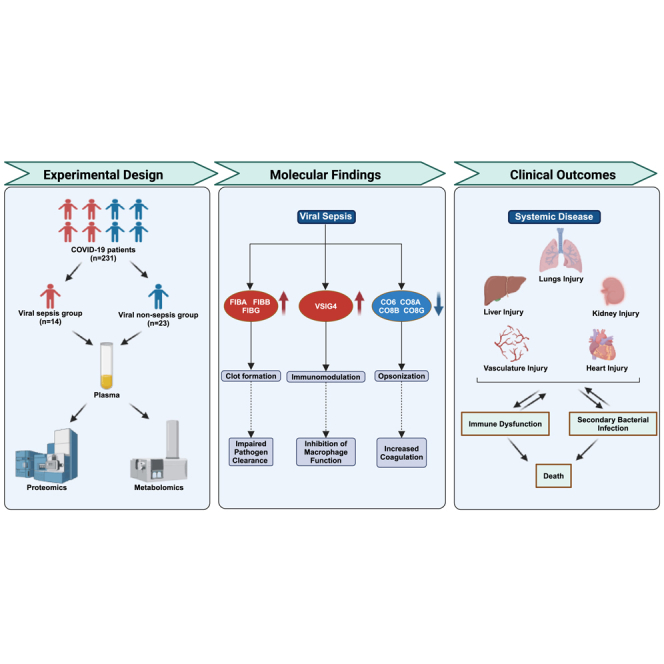

## Introduction

Sepsis is a pathological syndrome associated with multiple organ dysfunction, resulting in extensive morbidity and mortality. The World Health Organization estimates that globally, there are 50 million new diagnosis and more than 11 million deaths due to sepsis annually.^1^ Although the underlying etiologies remain diverse, sepsis is defined as a dysregulated host immune response that proves detrimental to the host. Much of the existing literature focused on bacterial pathogens as a cause of sepsis, even in cases where no bacterial pathogens are found to be associated with underlying disease. In contrast, viral sepsis has received limited attention in sepsis literature, partly due to the challenges in viral detection. However, with the advancement in the detection of viral pathogens, viral infections are increasingly recognized as a causative agent for sepsis. The emergence of COVID-19 pandemic has further emphasized the role of viral infections in causing systemic diseases, including sepsis.

COVID-19 has emerged as one of the most devastating pandemics of modern times, infecting a significant population of the world and causing millions of hospitalizations and deaths.^2^ Clinical and translational research over the last three years have illuminated key aspects of COVID-19 pathologies, including both pulmonary and extrapulmonary manifestations.^3^^,^^4^^,^^5^ The extrapulmonary manifestations of COVID-19 have been attributed to multiple factors including viremia, cytokine storm, and the persistent activation of immune cells.^6^^,^^7^ Recent studies have started to recognize extensive presence of sepsis in COVID-19, which has been associated with elevated mortality rates, even when compared to sepsis of other etiologies including abdominal, pulmonary, and urogenital origins.^8^ In COVID-19, the prevalence of sepsis in intensive care unit (ICU) subjects can be as high as 80%, whereas the prevalence is approximately 33% in those hospitalized in general ward.^9^ These findings underscore the critical need for a deeper understanding of sepsis caused by COVID-19, aiming to enhance the identification and management of such cases and thereby mitigate the excessive mortality associated with them.

Here, utilizing data from three hospitals in China, we identified cases of viral sepsis caused by COVID-19. We delineated unique clinical and laboratory characteristics of those with viral sepsis compared to COVID-19 subjects without sepsis. Furthermore, we conducted proteomic and metabolomic analysis using plasma samples from a subset of individuals with COVID-19 sepsis and those with COVID-19 without sepsis, aiming to demonstrate a distinctive molecular signature indicating specific pathological mechanisms of viral sepsis in COVID-19.

## Result

### Cohort characteristics, clinical characteristics, and prognosis

**Figure 1:**
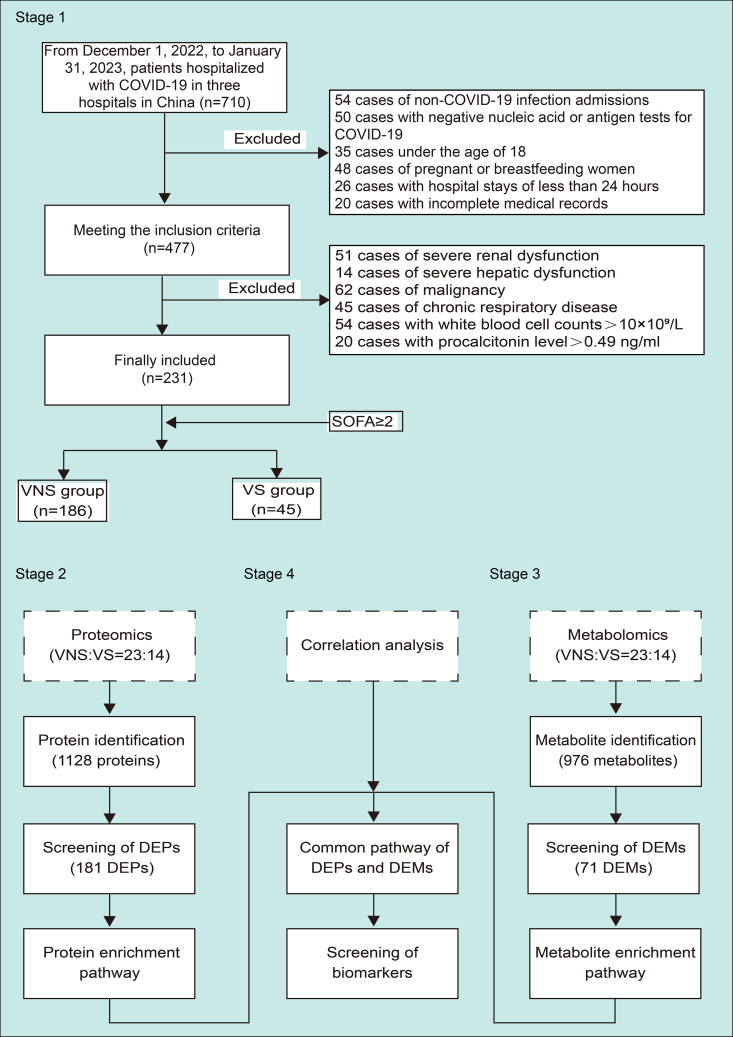
The flow chart demonstrating the study design

A total of 710 patients were admitted to three hospitals in China (The Seventh Medical Center of the General Hospital of the People’s Liberation Army of China, The Second Hospital of Hebei Medical University, and Tsinghua Chang Gung Hospital) between December 1, 2022 and January 31, 2023. Omicron strain of the coronavirus was the most prevalent strain in China during this outbreak.^10^ Exclusion criteria, such as non-COVID-19 etiology, a negative COVID-19 test, breastfeeding mothers, hospitalization less than 24 h, or incomplete medical records, led to the exclusion of 233 patients. Additionally, patients with a history of severe renal or hepatic dysfunctions, malignancy, and chronic lung diseases were excluded. White blood cell (WBC) counts >10×10^9^/L or procalcitonin levels of >0.49 ng/mL at the time of admission to hospital were excluded to rule out presence of secondary bacterial infection at the presentation. Finally, 231 patients were included in the study, and based on the Sequential Organ Failure Assessment (SOFA) score, they were classified into either the COVID-19 sepsis group or VS (SOFA score ≥2) or COVID-19 non-sepsis (VNS) group (SOFA score <2). The study design is illustrated in Figure 1.

### Clinical characteristics of patient cohort

Demographics and clinical characteristics of VS and VNS subjects are indicated in the Table 1. Subjects in the VS group were significantly older than VNS group (75 vs. 83 years). There was no statistical difference between sex and comorbidities present in the two groups. Patients with viral sepsis had a significantly higher incidence of disease severity classified as “critical” (55% vs. 6%, *p* < 0.001) based on the guidelines.^11^ A significantly higher fraction of VS group had acute respiratory distress syndrome [ARDS] (73% vs. 15%, *p* < 0.001) and respiratory failure (62% vs. 5%, *p* < 0.001). The high rate of respiratory failure led to increased requirements for invasive (0 vs. 33.3%, *p* < 0.001) and non-invasive (0 vs. 15.5%, *p* < 0.001) ventilation in the VS group. VS group also exhibited a significant increase in cardiac, liver, and kidney injuries consistent with phenotypes observed in sepsis.^12^^,^^13^ Furthermore, in concordance with sepsis-mediated immunosuppression,^14^ a significant proportion of the VS group had subsequent secondary bacterial (55% vs. 16%) or fungal (15.6% vs. 2.2%) infections compared to the VNS group. Overall, the VS group had significantly more ICU admissions, longer stays, and higher overall hospitalization costs compared to the VNS group. Consistent with the high mortality rates associated with sepsis, we observed a 42% mortality rate in the VS group compared to a 1.6% mortality rate in the VNS group on day 28 post-infection. The mortality continued even weeks after the diagnosis of sepsis in the VS group (Figure S1).

**Table 1:** Demographics and clinical characteristics of patients with viral sepsis and viral non sepsis groups with COVID-19

	All (*n* = 231)	VNS group (*n* = 186)	VS group (*n* = 45)	*P*
Age/[years，*Md*(IQR)]	76.00 (67.00，85.00)	75.00 (64.00, 84.00)	83.0 (72.00, 90.00)	＜0.001
Gender/(n，%)				
Male	132 (57.1)	107 (57.5)	25 (55.6)	0.810
Female	99 (42.9)	79 (42.4)	20 (44.4)	
Classification of COVID-19/(n，%)				
Severe condition	79 (34.2)	41 (22.0)	38 (84.4)	＜0.001
Non-severe condition	152 (65.8)	145 (78.0)	7 (15.6)	
Underlying Conditions/(n，%)				
Hypertension	128 (55.4)	100 (53.7)	28 (62.2)	0.306
Type 2 diabetes	67 (29.0)	55 (29.6)	12 (26.7)	0.700
Coronary heart disease	86 (37.2)	62 (33.3)	24 (53.3)	0.013
Cerebrovascular disease	46 (19.9)	35 (18.8)	11 (24.4)	0.396
Smoking History/(n，%)	31 (13.4)	27 (14.5)	4 (8.9)	0.320
Alcohol History/(n，%)	23 (10.0)	23 (12.4)	0 (0)	0.027
Time from Symptoms to Admission/[d，Md(IQR)]	10.00 (6.00，14.00)	10.00 (5.00, 14.00)	10.00 (7.00, 12.00)	0.650
Length of Hospital Stay/[d，Md(IQR)]	12.00 (7.00，16.00)	12.00 (7.75, 16.00)	11.00 (6.00, 20.50)	0.965
Hospitalization Cost/[10,000 RMB，Md(IQR)]	1.54 (0.81，2.73)	1.41 (0.81, 2.52)	2.48 (0.83, 7.81)	＜0.001
ICU Admission/[d，Md(IQR)]	16 (6.9)	5 (2.7)	11 (24.4)	＜0.001
ICU Length of Stay/[d，Md(IQR)]	11.0 (5.25, 16.0)	10.50 (7.75, 13.75)	11.50 (3.75, 16.75)	＜0.001
BMI (kg·m^−2^， $\bar{X}$ ± s)	24.32 ± 3.44	24.39 ± 3.21	24.03 ± 4.36	0.654
Treatment/(n，%)				
High-flow nasal oxygen	53 (22.9)	42 (22.6)	11 ( (24.4)	0.790
Invasive mechanical ventilation	15 (6.5)	0 (0)	15 (33.3)	＜0.001
Non-invasive mechanical ventilation	7 (3.0)	0 (0)	7 (15.5)	＜0.001
Complications/(n，%)				
ARDS	61 (26.4)	28 (15.1)	33 (73.3)	＜0.001
Respiratory failure	38 (16.5)	10 (5.4)	28 (62.2)	＜0.001
Bacterial pneumonia	55 (23.8)	30 (16.1)	25 (55.6)	＜0.001
Fungal pneumonia	11 (4.8)	4 (2.2)	7 (15.6)	＜0.001
Cardiac injury	28 ( (12.1)	9 (4.8)	19 (42.2)	＜0.001
Liver injury	30 (13.0)	15 (8.1)	15 (33.3)	＜0.001
Kidney injury	17 (7.4)	5 (2.7)	12 (26.7)	＜0.001
Lower limb thrombosis	4 (1.7)	1 (0.5)	3 (6.7)	0.024
Cerebral infarction	8 (3.5)	7 (3.7)	1 (2.2)	0.958
Myocardial infarction	4 (1.7)	1 (0.5)	3 (6.7)	0.024
Prognosis/(n，%)				
Survival	209 (90.5)	183 (98.4)	26 (57.8)	＜0.001
Death	22 (9.5)	3 (1.6)	19 (42.2)	

### Laboratory characteristics of viral sepsis in COVID-19

The laboratory measurements of the patients comparing the VS with VNS group are demonstrated in Table 2. The data are from the first blood sample collected from these subjects indicated in Table 1 within 24 h of admission. Although, there was no difference in the overall WBC counts, an increase in neutrophil and a decrease in lymphocyte and monocyte populations was observed in the VS group compared to the VNS group. Consistitent with the inflammatory phenotype in the VNS group, we observed a significant elevation of C-reactive protein (CRP) (20.95 vs. 64.80 mg/L, *p* < 0.001). A decrease in platelet counts was also observed in the VS sepsis group (216 vs. 143 ×10^9^/L, *p* < 0.001). Furthermore, markers of the elevated coagulation pathway, such as elevated D-dimer, were significantly increased in the VS group. Laboratory parameters indicated the presence of multi-organ injuries, including liver (alanine aminotransferase [ALT] and aspartate aminotransferase [AST]), cardiac (troponin), and muscle injury (myoglobin). Although, the overall levels of procalcitonin were low, given our exclusion criteria, the VS group still had significantly high levels of procalcitonin compared to the non-sepsis subjects (0.04 vs. 0.17 ng/mL).

**Table 2:** Laboratory findings of patients populations with VS and VNS groups (Mean ± SD) or Md (IQR) or (n, %)

	All (*n* = 231)	VNS group (*n* = 186)	VS group (*n* = 45)	*P*
White blood cell count/(L^−1^，×10^9^)	6.08 ± 1.94	6.05 ± 1.76	6.20 ± 2.59	0.716
Neutrophil percentage/(%)	72.30 (61.70，81.20)	71.20 (60.08, 79.03)	78.70 (69.50, 87.55)	˂0.001
Lymphocyte percentage/(%)	18.20 (11.70，27.40)	19.80 (12.20, 28.73)	13.70 (8.55, 20.65)	0.003
Monocyte percentage/(%)	7.10 (4.80，9.20)	7.40 (5.50, 9.25)	4.70 (2.95, 7.65)	0.001
Red blood cell count/(L^−1^, ×10^12^)	4.14 (3.73，4.50)	4.15 (3.76, 4.53)	4.13 (3.57, 4.42)	0.291
Hemoglobin/(g·L^−1^)	125.66 ± 17.90	126.27 ± 17.05	123.13 ± 21.08	0.292
Platelet count/(L^−1^, ×10^9^)	208.00 (161.00，255.00)	216.00 (167.75,263.25)	143.00 (96.00,201.50)	˂0.001
Alanine aminotransferase/(U·L^−1^)	22.00 (13.38，36.00)	22.00 (13.88, 36.00)	21.50 (12.25, 40.65)	0.704
Aspartate aminotransferase/(U·L-1)	24.00 (18.00, 35.00)	23.00 (18.00, 32.50)	34.00 (20.50, 49.00)	0.005
Total bilirubin/(μmol·L^−1^)	10.17 (7.15，13.90)	10.05 (6.78, 12.83)	11.98 (8.18, 17.32)	0.004
Direct bilirubin/(μmol·L^−1^)	4.35 (3.10，6.05)	4.16 (3.00，5.66)	5.67 (3.82, 8.81)	˂0.001
Albumin/(g·L^−1^)	36.22 ± 5.02	36.67 ± 4.76	34.34 ± 5.67	0.005
Blood glucose/(mmol·L^−1^)	6.45 (5.45, 8.32)	6.26 (5.22, 8.21)	7.66 (6.18，8.61)	0.011
Blood urea nitrogen/(mmol·L^−1^)	5.65 (4.12, 7.51)	5.50 (4.12, 6.88)	7.15 (4.68, 9.32)	0.003
Creatinine/(μmol·L^−1^)	69.85 (55.90, 83.00)	68.80 (55.15, 81.05)	77.10 (57.18, 93.25)	0.069
Uric acid/ (μmol·L^−1^)	265.96 ± 102.76	266.13 ± 100.60	265.32 ± 112.12	0.963
Lactate dehydrogenase/(U·L^−1^)	223.00 (181.00, 273.50)	211.00 (175.25，265.50)	259.00 (195.00, 399.00)	＜0.001
Creatine kinase/(U·L^−1^)	68.50 (40.25, 117.50)	65.00 (40.00, 98.00)	91.00 (43.50, 245.50)	0.020
Creatine kinase-MB/(U·L^−1^)	11.65 (8.53, 15.08)	11.80 (8.48, 15.00)	11.25 (8.68, 17.53)	0.647
Total cholesterol/(mmol·L^−1^)	3.69 (3.16, 4.25)	3.78 (3.25, 4.41)	3.48 (2.89, 3.96)	0.025
Triglycerides/(mmol·L^−1^)	1.11 (0.82, 1.38)	1.08 (0.82, 1.39)	1.16 (0.80, 1.37)	0.528
Homocysteine/(μmol·L^−1^)	13.90 (10.00, 19.15)	12.95 (9.93, 17.75)	17.80 ( (10.48, 22.40)	0.009
Lactic acid/(mmol·L^−1^)	1.50 (1.10, 2.10)	1.50 (1.00, 2.00)	1.60 (1.30, 2.40)	0.011
Cystatin C/(mg·L^−1^)	1.14 (0.93, 1.45)	1.10 (0.92, 1.39)	1.29 (1.03, 1.54)	0.021
Myoglobin/(ng·ml^−1^)	63.00 (44.00, 126.00)	57.85 (43.00, 109.08)	144.00 (61.00, 436.00)	˂0.001
Troponin I/(ng·ml^−1^)	0.54 (0.40，0.67)	0.53 (0.04, 0.65)	0.57 (0.48, 0.80)	0.011
N-terminal pro-brain Natriuretic peptide/(pg·ml^−1^)	268.50 (107.00, 980.25)	219.50 (94.00, 646.50)	879.00 (221.00, 1689.75)	0.001
C-reactive protein/(mg·L^−1^)	30.40 (6.70, 81.80)	20.95 (5.53, 74.78)	64.80 (27.25, 116.00)	˂0.001
Erythrocyte sedimentation rate/(mm·h^−1^)	42.50 (18.25, 70.00)	36.00 (16.00, 65.00)	64.00 (33.00, 80.00)	0.042
Procalcitonin/(ng·ml^−1^)	0.06 (0.01, 0.16)	0.04 (0.01, 0.11)	0.17 (0.07, 0.28)	˂0.001
Ferritin/(ng·ml^−1^)	367.20 (217.10, 717.10)	305.60 (171.70, 631.20)	760.30 (501.25, 928.43)	0.002
Prothrombin time/(s)	12.10 (11.40, 13.00)	12.20 (11.30, 12.90)	12.10 (11.45, 13.48)	0.685
Prothrombin activity/(%)	87.62 ± 12.60	88.22 ± 12.55	85.23 ± 12.65	0.159
INR	1.09 (1.03, 1.15)	1.07 (1.02, 1.15)	1.10 (1.04, 1.23)	0.104
APTT/(s)	31.50 (29.18, 34.33)	31.40 (28.90, 34.10)	32.50 (30.60, 35.05)	0.039
Fibrinogen/(g·L-1)	4.02 (3.35, 4.87)	3.99 (3.34, 4.94)	4.06 (3.59, 4.77)	0.908
Thrombin time/(s)	13.40 (12.10, 14.60)	13.40 (12.10, 14.53)	13.65 (12.30, 15.30)	0.425
D-dimer/(mg·L-1)	0.25 (0.11, 0.58)	0.23 (0.10, 0.44)	0.32 (0.16, 1.35)	0.033

To gain a better understanding of the risk factors for viral sepsis, we performed univariate and multivariate logistic regression analyses for potential risk factors. Our analysis reveals several risk factors associated with the diagnosis of viral sepsis following COVID-19. The multivariate logistic regression analysis identified COVID-19 disease severity, lower platelet counts, and a diagnosis with ARDS as risk factors for viral sepsis (Table 3).

**Table 3:** Univariate and multivariate logistic regression analysis of risk factors for viral sepsis

	P	OR (95%CI)	P	OR (95%CI)
Age	0.002	1.043 (1.015，1.071)		
Platelet count	˂0.001	0.984 (0.977，0.99)	0.001	0.980 (0.969，0.992)
Total bilirubin	0.001	1.114 (1.044，1.187)		
Direct bilirubin	˂0.001	1.297 (1.14，1.475)		
Total protein	0.002	0.931 (0.89，0.975)		
Urea nitrogen	0.002	1.205 (1.073，1.352)		
LDH	˂0.001	1.008 (1.004，1.012)		
Myoglobin	˂0.001	1.005 (1.003，1.007)		
CRP	˂0.001	1.015 (1.007，1.023)		
COVID-19 severity	˂0.001	19.199 (7.983，46.172)	0.035	14.807 (1.484，147.750)
ARDS	˂0.001	15.518 (7.161，33.627)	0.014	15.898 (1.751，144.339)
Respiratory failure	˂0.001	28.988 (12.06，69.679)		

### Proteomic changes associated with viral sepsis

**Figure 2:**
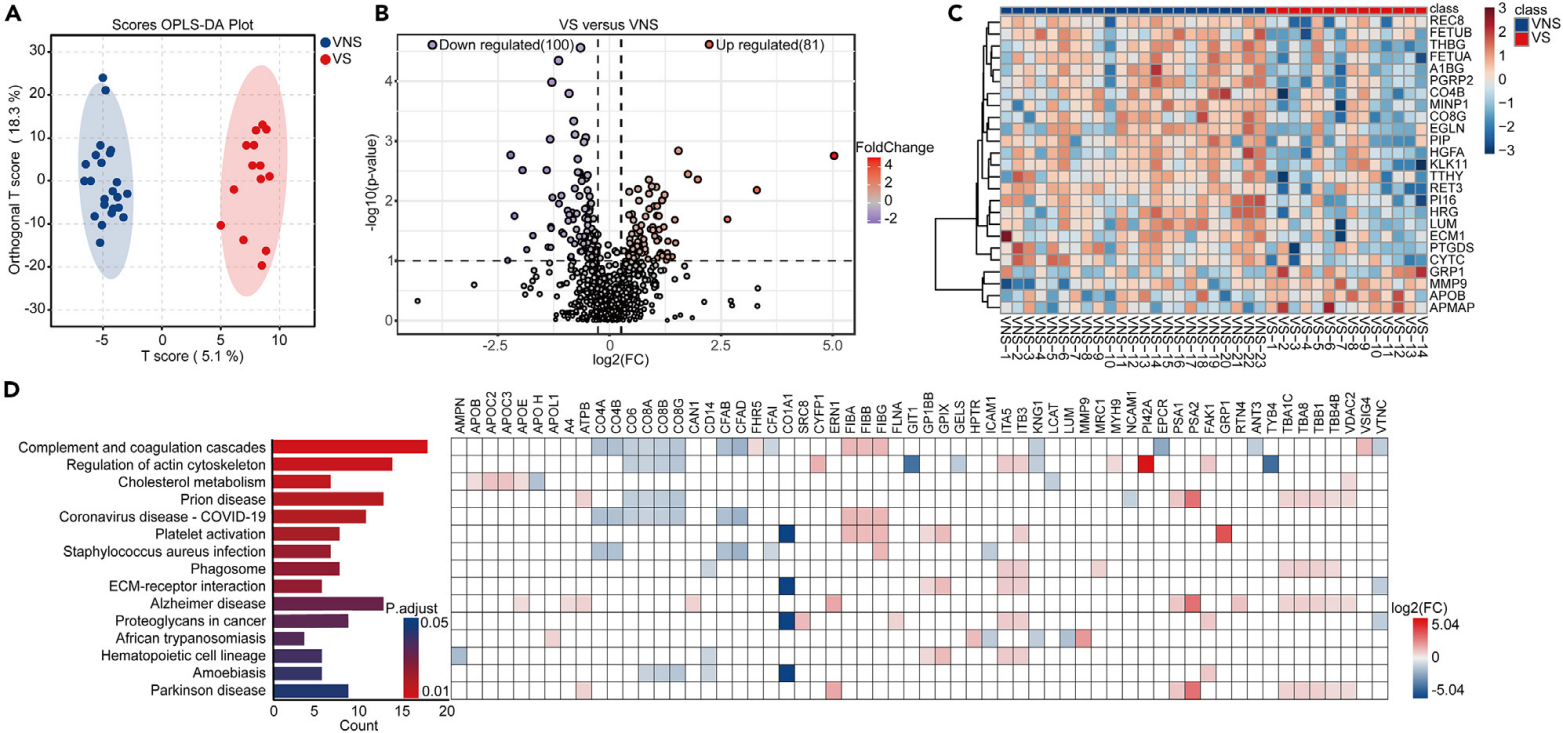
Proteomic changes in plasma samples of patients with viral sepsis compared to viral non sepsis Orthogonal partial least squares-discriminant analysis (OPLS-DA) of proteins between viral sepsis and viral non-sepsis groups (A). Volcano plot displaying differential expression of protein levels between viral sepsis (VS) group and viral non-sepsis (VNS) group (B). Heatmaps demonstrating top 25 differentially expressed proteins (C). The significantly enriched KEGG pathways and the corresponding differentially expressed proteins (DEPs). The color intensity demonstrates log2 fold changes and the color denotes up (red) or down (blue) (D).

To gain mechanistic insights into the pathophysiology of viral sepsis in an unbiased manner, we assessed the proteomic changes associated with viral sepsis. We identified the levels of a total 1,128 peptides from the plasma samples of 23 VNS and 14 VS patients. Clinical and laboratory analyses of these patients are shown in Table S1. Our data identified differentially expressed peptide levels between the two groups (Figure 2A). Volcano plots indicated differentially expressed proteins between the VS and VNS groups (Figure 2B). Among the top upregulated peptides in NVS group, we identified that FETUA (Fetuin-A), RET3 (retinol-binding protein 3), SHBG (sex-hormone-binding globulin), PGRP2 (peptidoglycan recognition protein 2), CYTC (cytochrome C), PI16 (peptidase inhibitor-16), HRG (histidine-rich glycoprotein), and FETUB (Fetuin-B) as well as PI42A (phosphatidylinositol 5-phosphate 4-kinase type-2 alpha), CELR2 (cadherin EGF LAG seven-pass G-type receptor 2), and RTN4 (reticulon 4) were the most upregulated proteins in the VS group (Figure 2C). To understand the biological significance of the proteomic changes observed in viral sepsis, we performed KEGG pathway analysis to identify pathways associated with these proteomic changes. Our analysis demonstrated that the “complement and coagulation pathway” was the top enriched pathway, whereas other significantly enriched pathways included “regulation of actin cytoskeleton,” “cholesterol pathway,” and “platelet activation pathway” (Figure 2D). The proteins associated with those pathways are indicated with their extent of changes.

We observed a significant upregulation of coagulation factors such as fibrinogen molecule components (FIBA, FIBB, and FIBG) in the VS group. The fibrinogen molecule, which is composed of two dimers of α, β, and γ chains (FIBA, FIBB, FIBG) form the blood clot upon cleavage by thrombin to fibrin, indicating elevated coagulation pathway in the viral sepsis group. Components of fibrinogen molecule have been shown to be elevated in the severe COVID-19 subjects and further elevated in non-survivors,^15^ indicating potential contribution in the disease severity. Further, a recent study has shown that elevated fibrinogen aggregates with amyloid were associated with sepsis phenotype and overall mortality in critically ill patients, supporting our data presented in this study.^16^ Additionally, there was an elevation of complement-related proteins such as FRH-5 (complement-factor-H-related protein 5) and VSIG4 (V-set and immunoglobulin domain-containing 4), which inhibits macrophage function,^17^ whereas a downregulation of complement proteins in the VS group including CO4A, CO4B, CO6, CO8A, CO8B, and CO8G. In the cholesterol pathway, we noted an upregulation of apolipoproteins including ApoB, ApoC2, ApoC3, and ApoE. In contrast, we observed a decrease in ApoH, which has known anti-coagulant activity through binding with thrombin to inhibit coagulation.^18^ Other markers that were significantly elevated in the VS group included those associated with platelet activation. These proteins include GP1BB (glycoprotein Ib platelet subunit beta) and glycoprotein IX (GPIX), both of which are associated with worse outcomes in the COVID-19.^19^^,^^20^ These proteomic changes indicate dysregulated coagulation, complement, and platelet activation as characteristic features of viral sepsis.

### Metabolomic changes associated with viral sepsis

**Figure 3:**
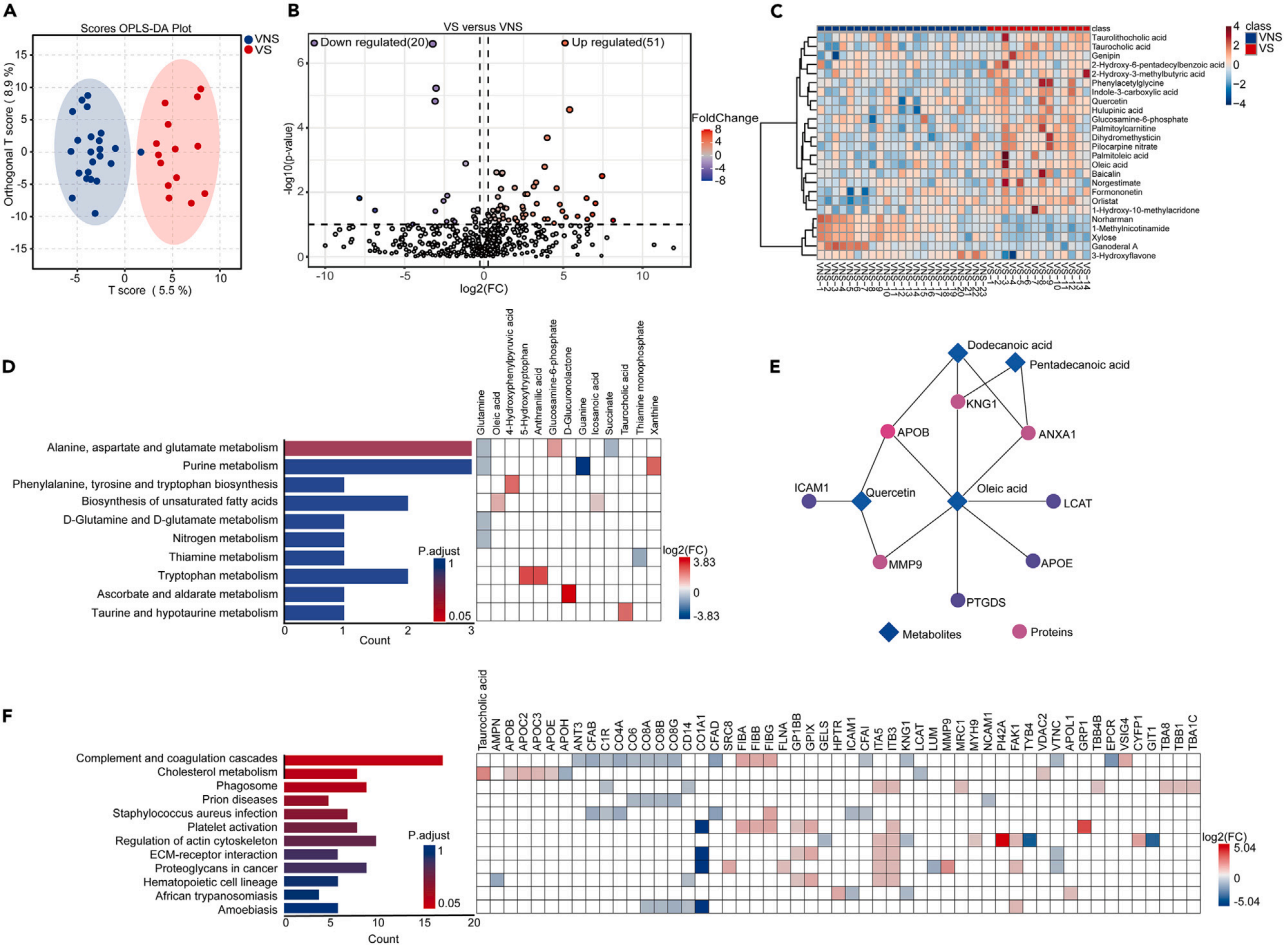
Metabolomic changes in plasma samples of patients with viral sepsis compared to viral non sepsis and combined pathway analysis with proteomic changes Orthogonal partial least squares-discriminant analysis (OPLS-DA) of metabolites between viral sepsis and viral non-sepsis groups (A). Volcano plot displaying differences in metabolite levels between viral sepsis group and viral non-sepsis groups (B). Heatmaps showing top 25 differentially expressed metabolites between viral sepsis and viral non sepsis groups (C). Significantly enriched pathways and the corresponding differentially expressed metabolites (DEMs) between viral sepsis and viral non sepsis group (D). The interaction between DEMs and DEPs (E). The significantly enriched pathways using combined DEPs and DEMs (F).

In order to gain mechanistic insights into pathophysiology of viral sepsis, we performed metabolomic analyses on the same plasma samples. Overall, the metabolomic profile between the two groups were different (Figure 3A). A total of 976 metabolites were identified where we observed both upregulated and downregulated metabolites among the two groups (Figures 3B and 3C). Among these metabolites, we focused on the host-derived metabolites to gain a better mechanistic understanding of the pathological response in viral sepsis. The top upregulated metabolite in the NVS group was 1-methylnicotinamide (1-MNA), known for its anti-inflammatory and anti-coagulant properties.^21^ 1-MNA supplementation has demonstrated a prolongation of lifespan in lower animals such as *Caenorhabditis elegans*.^22^ In the VS group, fatty acids, specifically oleic acid, were significantly upregulated compared to VNS group, consistent with previous studies indicating its elevation in COVID-19.^23^ KEEG pathway analysis revealed top-enriched pathways, including “alanine, aspartate, and glutamate metabolism,” “the purine metabolism,” as well as “biosynthesis of unsaturated fatty acids” (Figure 3D). Notably, these pathways are known to be associated with worse outcomes during COVID-19.^24^

The connectome analysis linked metabolites such as oleic acid with others controlling the cholesterol pathway, as well as those associated with matrix, such as matrix metalloproteinase 9 (MMP9) (Figure 3E). The combined pathway analysis of proteins and metabolites confirmed dysregulation in the complement and coagulation pathway as a key pathology in the viral sepsis group (Figure 3F).

### Predicting the diagnosis of viral sepsis through specific multi-omics changes

**Figure 4:**
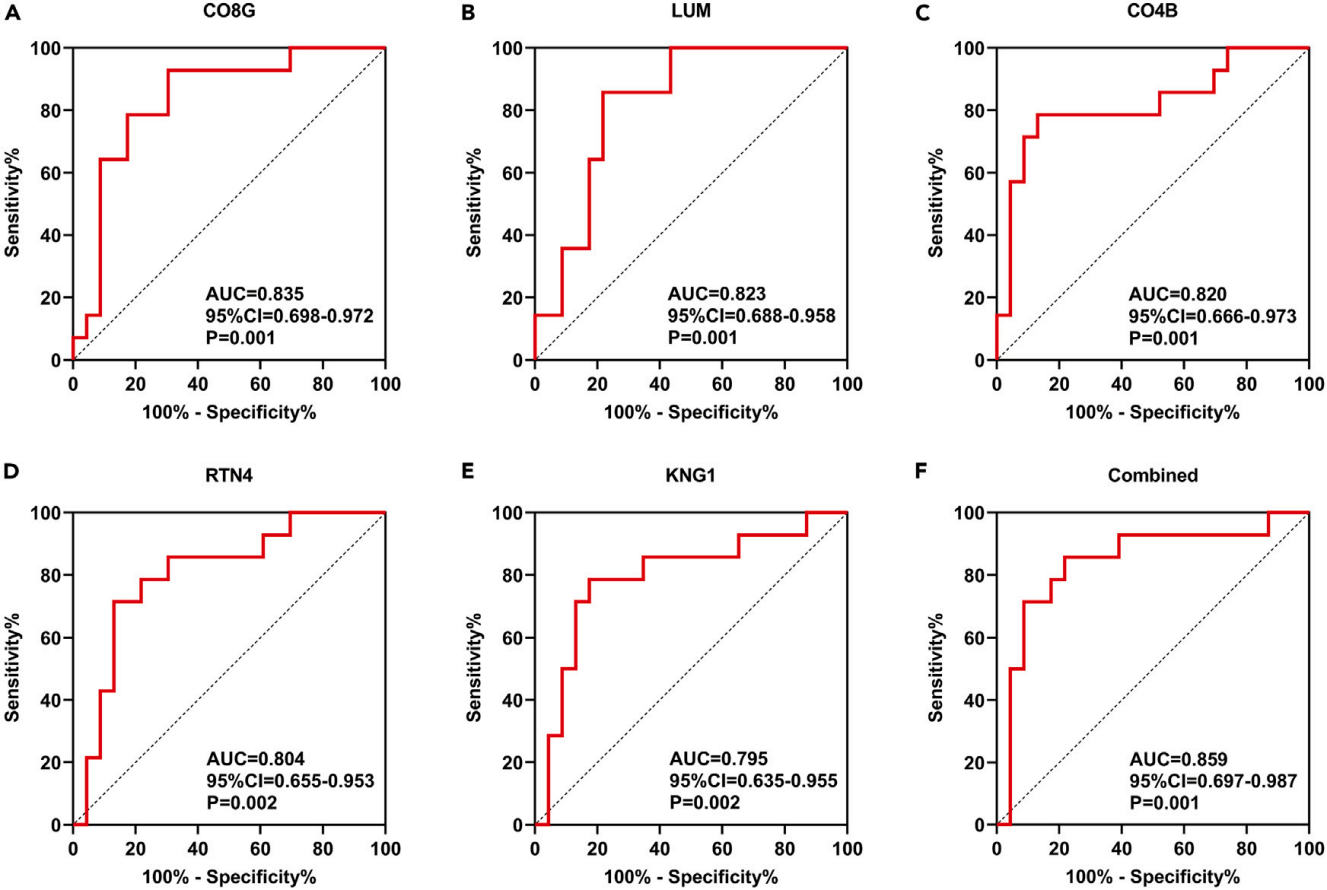
Molecular biomarkers of viral sepsis in COVID-19 (A–E) The receiver operating curves (ROC) of top five DEPs or DEMs (A–E) and a combined ROC (F) in predicting viral sepsis. P values are indicated on the graph.

After identifying distinct metabolomic and proteomic alternations, we sought to determine if these metabolomic changes have any diagnostic value for viral sepsis in COVID-19. We initially assessed the predictive value of individual markers, including proteomic or metabolomic indicators. We identified a total of 39 markers, each with an area under the curve (AUC) of 0.70 (Table S2). Among these markers, we selected top five biomarkers associated with the onset of viral sepsis. Our data show that complement C4b, C8g, Lum, RTN4, and KNG-1 were among the top proteins and metabolites with predictive values (Figures 4A–4E). Combining these five markers, we achieved an AUC of 0.859, indicating the diagnostic value of these markers for viral sepsis in COVID-19.

## Discussion

Our study highlights key pathological changes associated with COVID-19 sepsis. Utilizing proteomic and metabolomic analyses, our data identified that dysregulated coagulation and complement pathways are crucial determinants of COVID-19 sepsis. Both complement dysregulation and enhanced coagulation are hallmarks of disease severity in the COVID-19.^25^^,^^26^ Our data show that viral sepsis was associated with significant decrease in the components of classical complement system including CO4A, CO4B, as well as terminal complement components CO6, CO8A, CO8B, and CO8G, which sheds light into pathological and potentially immune evasion phenotype of SARS-CoV-2. In the coagulation pathway, we observed significant upregulation of fibrinogen molecule components including fibrinogen A, B, and G. This was supported by laboratory measurement demonstrating elevated levels of D-dimer in VS group.

These mechanisms have been shown by other viruses such as flaviviruses, which cleave C4 protein to block the neutralization by the complement system.^27^ These data may also shed light into the pathological mechanisms such as susceptibility to secondary infections including both bacterial and fungal infections. A dysregulated complement system, especially a decrease in many of the complement proteins, can render the host susceptible to secondary infections as observed in our viral sepsis cohort. The complement system is an essential component of phagocytosis-mediated clearance of the bacterial pathogens by professional phagocytes such as macrophages, monocytes, and neutrophils.^28^^,^^29^ Phagocytosis-independent antibacterial mechanisms of complement have been reported.^30^ Genetic defects in the complement system have been associated with increased susceptibility to bacterial and fungal infections.^31^ A strong complement response has been associated with improved survival in critically ill subjects^32^; however, it remains unclear whether it was due to protection from secondary bacterial infections.

Consistent with our data, secondary bacterial infections in COVID-19 significantly contribute to prolonged disease and adverse outcomes, especially in severe cases.^33^^,^^34^ Sepsis-mediated immune dysregulation increases host susceptibility to secondary infections by bacterial and fungal pathogens, complicating disease prognosis. Our data provide novel mechanistic insights into this impaired host immunity during viral sepsis. As a consequence of the impaired host immunity, the secondary infections potentially contribute to the persistent risk of mortality even weeks after initial diagnosis of sepsis observed in our cohort.

Sepsis remains a leading cause of the death worldwide and have no clinically effective therapy available to reduce mortality. The role of viral pathogens as a mediator of sepsis is less recognized in the literature, and mortality estimates of viral sepsis remain heterogeneous. Prior to the onset of COVID-19, only a few studies have investigated the prevalence of viral sepsis. Early evidence of viruses contributing to sepsis emerged from observations of seasonal variations in sepsis cases, where an increase in cases correlated with the peak viral seasons.^35^ A study conducted in Spain investigated viral sepsis due to community-acquired pneumonia and estimated a one-year mortality of 8%.^36^

A study from three Asian countries identified a significant contribution of respiratory viruses to sepsis in both pediatric and adult patients. This study estimated the overall mortality due to sepsis to 13% (South Asian study). It is not surprising that the role of viruses as a cause of sepsis was more appreciated in children than in adults, given the frequent and often severe viral infections in the pediatric populations.^37^^,^^38^ However, it is highly likely that all these studies underestimate the true prevalence of viral sepsis due to limited testing of viral pathogens beyond common viruses.

The emergence of COVID-19 pandemic significantly increased our understanding of viral pathogenesis; however, its contribution to the sepsis remains incompletely understood. Early efforts to identify the risk factors for viral sepsis in COVID-19 identified advanced age and comorbidities as the risk factors for viral sepsis.^39^ Nevertheless, this study had only a few subjects with sepsis defined as SOFA≥2. Our study utilized all the subjects who tested positive for COVID-19 to identify the characteristics of sepsis in COVID-19. Since our control population was also positive for COVID-19, it allowed us to discriminate between the beneficial host immune response required to deal with COVID-19 and those that are pathogenic to the host. This sets our study apart from the proteomic analyses performed in other studies that either compared sepsis due to pneumonia with COVID-19,^40^ SARS-CoV-2 viremia in absence of sepsis phenotype,^41^ or compared different disease severity of COVID-19.

Overall, we present clinical and laboratory data to illustrate viral sepsis as a key pathological event that contributes to the disease severity and overall prognosis. Our proteomic and metabolomics data shed mechanistic insights into the pathophysiology of viral sepsis in COVID-19. These findings provide potential biomarkers and therapeutic targets for detecting and treating viral sepsis.

### Limitations of the study

We have a few limitations of the study. First, our study is limited by its retrospective nature; therefore, these findings require validation in future prospective studies, including the validation of our biomarker analyses in larger cohorts. Furthermore, our study provides a potential link between the dysregulation of complement and coagulation pathways and viral sepsis in COVID-19; however, it remains to be known whether similar dysregulation is observed in viral sepsis of non-COVID-19 origin. Finally, our study was exclusively conducted within one racial group, which limits our ability to identify any racial differences that may exist in viral sepsis in COVID-19 and associated proteomic and metabolomic changes.

## Quantification and statistical analysis

Statistical analysis was conducted using SPSS 27.0 and GraphPad Prism 9.0 software. Categorical data were expressed as counts and percentages, and between-group comparisons were carried out using the chi-square test. Normally distributed continuous data were presented as mean ± standard deviation (SD), and between-group comparisons were performed using the independent samples t-test. Non-normally distributed data were presented as medians with interquartile ranges (IQR), and between-group comparisons were made using the Mann-Whitney U test. Univariate and multivariate logistic regression analyses were employed to explore the risk factors associated with VS. A significance level of *p* < 0.05 was considered statistically significant.

## STAR★Methods

### Key resources table

**Table:** 

REAGENT or RESOURCE	SOURCE	IDENTIFIER
**Biological samples**		

Human plasma	This paper	N/A

**Chemicals, peptides, and recombinant proteins**		

Acetonitrile	Fisher Chemical	Cat#75-05-8
Formic Acid	Sigma-Aldrich	Cat#64-18-6
Ammonium Bicarbonate	Sigma-Aldrich	Cat#1066-33-7
Dithiothreitol	Sigma-Aldrich	Cat#3483-12-3
Iodoacetamide	Sigma-Aldrich	Cat#144-48-9
Trypsin	Promega	Cat#9002-07-7
Acetone	Sigma-Aldrich	Cat#67-64-1
Ultra-pure water	This paper	N/A
Formic Acid	Fisher Chemical	Cat#64-18-6
Methanol	Fisher Chemical	Cat#67-56-1

**Deposited data**		

Mass spectrometry proteomics data	This paper	https://proteomecentral.proteomexchange.org; ID：PXD050303
Untargeted metabolomic data	This paper	https://www.ebi.ac.uk/metabolights/MTBLS9664

**Software and algorithms**		

GraphPad Prism 9.0	GraphPad Software, Inc.	https://www.graphpad.com
R	https://cran.r-project.org	V4.3.1
Biorender	Biorender	https://www.biorender.com/
Proteome Discoverer	Thermo Fisher Scientific (USA)	RRID:SCR_014477
UniProt	https://www.uniprot.org/	RRID:SCR_002380
MetaboAnalyst	https://www.metaboanalyst.ca/	RRID:SCR_015539
BiocManager package	https://bioconductor.org/	N/A
STRING	http://string.embl.de/	RRID:SCR_005223
MS-DIAL	http://prime.psc.riken.jp/compms/msdial/main.html	RRID:SCR_023076
IBM SPSS Statistics	https://www.ibm.com/products/spss-statistics	RRID:SCR_016479
HMDB	http://www.hmdb.ca	RRID:SCR_007712

**Other**		

Vortex Mixer	Changzhou Enpei Instrument Manufacturing Co., Ltd	NP-30S
Mini Centrifuge	SCILOGEX	D1008E
High-Speed Refrigerated Centrifuge	SIGMA	1-15K
Analytical Balance	Mettler Toledo Instruments (Shanghai) Co., Ltd	AL104
Vacuum Centrifugal Concentrator	Thermo Fisher Scientific (USA)	SPD121P-230
Electrospray Ionization Hybrid Ion Trap-Orbitrap Mass Spectrometer	Thermo Fisher Scientific (USA)	Orbitrap Fusion™ Lumos™ Tribrid™ Mass Spectrometer
High-Performance Liquid Chromatograph	Thermo Fisher Scientific (USA)	Vanquish
High-Performance Liquid Chromatograph	Thermo Fisher Scientific (USA)	Ultimate 3000
Mass Spectrometer	AB SCIEX™	TripleTOF5600+
Vacuum Centrifugal Concentrator	Eppendorf (Germany)	Concentrator plus
Analytical Balance	Sartorius (Switzerland)	Sartorius BP211d
Low-Temperature High-Speed Centrifuge	Beckman Coulter (USA)	Microfuge 22R Centrifuge
Mini Centrifuge	Scilogex (USA)	D1008
Vortex Mixer	Scilogex (USA)	MX-S

### Resource availability

#### Lead contact

Further information and requests for resources and reagents should be directed to the lead contact Professor De Chang at (changde@301hospital.com.cn).

#### Materials availability

This study did not generate new reagents.

#### Data and code availability

Mass spectrometry proteomics data and Untargeted metabolomic data have been deposited and are publicly available as of the date of publication. The access URLs for the datasets are listed in the key resources table.

This paper does not report original code.

Any additional information required to reanalyze the data reported in this paper is available from the lead contact upon request.

### Experimental model and study participant details

In this retrospective study, we obtained clinical information through electronic medical record from 710 hospitalized patients with confirmed COVID-19 in three hospitals in China between December 2022 and January 2023. All patients enrolled in the study were of Asian descent. This study was approved by Institutional Committee of Seventh Medical Center of Chinese PLA Hospital and informed consent was obtained from the patients or their representatives. Patients were diagnosed with COVID-19 following the "Diagnosis and Treatment Plan for Novel Coronavirus Pneumonia (Trial Tenth Edition).^11^" Inclusion criteria: (1) SARS-CoV-2 infection (positive nucleic acid or antigen test); (2) age ≥18 years; (3) hospitalization duration >24 h; (4) complete medical history. Exclusion criteria included: (1) Pregnant or breastfeeding women; (2) malignant tumors; (3) history of severe liver or kidney failure; (4) history of chronic respiratory diseases; (5) blood routine: white blood cell count >10 × 10^9^/L, procalcitonin >0.49 ng/mL at the time of hospitalization to rule our prior bacterial infections.^42^^,^^43^ According to the Sepsis 3.0 diagnostic criteria, patients with a SOFA score ≥2 were classified into the Viral Sepsis group (VS), while those with SOFA scores <2 were classified into the Viral Non-Sepsis group (VNS). Ultimately, 231 COVID-19 patients were included in this retrospective study, with 45 in the VS group and 186 in the VNS group. Please refer to Figure 1 for details.

Patient data were systematically collected, including age, gender, time from symptom onset to hospital admission, underlying medical conditions, ICU admission, ICU length of stay, laboratory tests and imaging examinations at admission. Additionally, information on treatments, complications, and prognosis during hospitalization was documented. Severe liver dysfunction was defined as a Child-Pugh score >9. Severe kidney dysfunction was defined as a creatinine level >550 μmol/L. Severe cardiac dysfunction was defined as NYHA functional classification ≥ Class III. The diagnostic criteria for acute respiratory distress syndrome (ARDS) are based on the Berlin definition.

### Method details

#### Sample collection

Blood is collected from fasting COVID-19 patients within 24 h of admission using EDTA-containing tubes. The blood samples are then centrifuged at 500g for 15 min at 4°C to separate the plasma. Subsequently, the plasma is stored at −80°C.

#### Proteome analysis

Proteomic analyses were performed by Beijing Biotech-Pack Scientific (Beijing, China).^44^ Samples were prepared by first removing abundant proteins from plasma followed by protein digestion. The Nano LC-MS/MS analysis involving nano LC and mass spectrometry processes was performed to identify specific proteins. For mass spectrometry, an Orbitrap Fusion Lumos Tribrid Mass Spectrometer from Thermo Fisher Scientific, USA, was used. The raw mass spectrometry files were analyzed using Proteome Discoverer 3.0 to search the targeted protein database. Initially identified protein data were uploaded to UniProt (https://www.uniprot.org/) for comprehensive annotation, including retrieving protein names and other relevant information, thus laying the groundwork for further analysis. Upon completion of the annotation, MetaboAnalyst 6.0 (https://www.metaboanalyst.ca/) was utilized for data preprocessing. Statistical analyses included Orthogonal Partial Least Squares - Discriminant Analysis (orthoPLS-DA), Volcano Plot (setting a Fold Change (FC) threshold at 1.2 and a *p*-value threshold at 0.05), and Hierarchical Clustering Heatmaps. Finally, Gene Ontology (GO) and Kyoto Encyclopedia of Genes and Genomes (KEGG) analyses of differentially expressed proteins were performed using R (version 4.3.1) with the BiocManager package, and results were visualized using the enrichplot and ggplot2 packages.

#### Metabolomic analysis

In the sample pre-treatment process for metabolomic extraction, 100 μL of the sample was taken and mixed with 300 μL of acetonitrile. This mixture was then vortexed, followed by ultrasonication for 30 min. Subsequently, it was centrifuged at 12,000 rpm and 4°C for 10 min, after which the supernatant was collected for analysis. For the LC-MS/MS detection, the liquid chromatography conditions were as follows: A Sepax GP-C18 Column (1.8 μm 120 Å 2.1 mm∗150 mm) was used; the column temperature was maintained at 40°C; the mobile phase A consisted of 0.1% formic acid; the mobile phase B was 100% ACN.

In the mass spectrometry analysis, detection was conducted using electrospray ionization (ESI) in both positive and negative ion modes. For database retrieval, the wiff files collected by the mass spectrometer were pre-processed using MS-DIAL 4.70 software. Initially, the metabolite data identified through analytical methods were referenced against the Human Metabolome Database (HMDB) (https://hmdb.ca/) for accurate annotation and confirmation of human metabolites, setting the stage for further analysis. Subsequent to this metabolite annotation, MetaboAnalyst 6.0 was employed for data preprocessing, involving steps such as data filtering with a variance filter (Interquartile range (IQR) and a 0% filtering percentage), sample normalization by sum, log transformation (base 10) for data transformation, and auto scaling for data scaling, wherein each variable was mean-centered and divided by its standard deviation. Statistical analysis was performed using Orthogonal Partial Least Squares - Discriminant Analysis (orthoPLS-DA) and Volcano Plots, setting an FC threshold at 1.2 and a *p*-value threshold at 0.05. This was complemented by Hierarchical Clustering Heatmaps analysis. Finally, an enrichment pathway analysis was conducted on the differentially expressed metabolites.

#### Integrated pathway analysis of proteomics and metabolomics

The joint pathway analysis conducted using MetaboAnalyst 6.0 enables the visualization of significant proteins and metabolites enriched in specific pathways. Network analysis in MetaboAnalyst 6.0 was performed utilizing the protein-metabolite interaction network mode, among other available modes, facilitating a comprehensive understanding of the complex interactions between different biological entities.

#### Identification of potential biomarker metabolites and proteins for viral sepsis

Metabolites and proteins were analyzed for biomarker potential in MetaboAnalyst 6.0. Classical univariate Receiver Operating Characteristic (ROC) curves were generated to determine Area Under the Curve (AUC) and 95% confidence intervals (CIs) for individual proteins or metabolites. FC was calculated dividing normalized abundance of VS subject by normalized abundance of VNS subject. We set a threshold for the AUC of the ROC curve at 0.70 and considered metabolites and proteins with an FC in the range of −3.867 to 1.278 as potential biomarkers. Visualizing DEPs and DEMs was performed using GraphPad Prism 9.0 software.
