# Supplementary material for: Clinical features and multiomics profiles indicate coagulation and platelet dysfunction in COVID-19 viral sepsis
Source: iScience. 2024 May 25;27(6):110110. doi: 10.1016/j.isci.2024.110110 (PMC11225851; doi:10.1016/j.isci.2024.110110)
Supplement: Document S1. Figure S1, Tables S1, and S2 [file mmc1.pdf]

**iScience, Volume *27***

## Supplemental information

# Clinical features and multiomics profiles indicate coagulation and platelet dysfunction in COVID-19 viral sepsis

**Zhiqing Xiao, Minggui Lin, Ning Song, Xue Wu, Jingyu Hou, Lili Wang, XinLun Tian, Chunge An, Charles S. Dela Cruz, Lokesh Sharma, and De Chang**

**Supplemental information**

Figure S1. Survival analysis of those with viral sepsis (VS) and viral non sepsis group (VNS), related to Table 1.

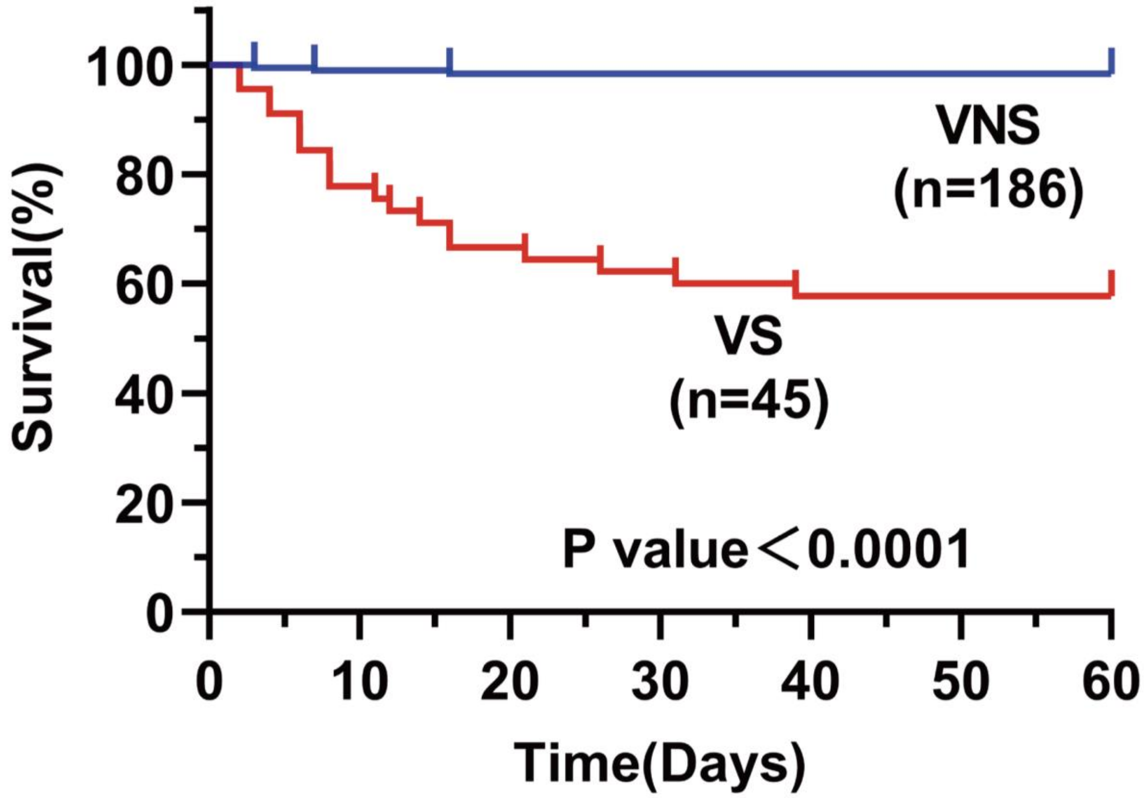

Table S1. Demographics and clinical characteristics of patients with viral sepsis (VS) and viral non sepsis (VNS) groups with COVID-19 whose sample were sent to proteomic and metabolomic analyses. Related to Figures 2 and 3.

| | All（n=37） | VNS group（n=23） | VS group（n=14） | *P* |
|---|---|---|---|---|
| **Age/[years，$\overline{X}$ ± s]** | 78.19±9.22 | 78.78±10.28 | 77.21±7.40 | 0.622 |
| **Gender/(n，%)** | | | | |
| **Male** | 21（56.76） | 13（56.52） | 8（57.14） | 0.970 |
| **Female** | 16（43.24） | 10（43.48） | 6（42.86） | |
| **classification of COVID-19/(n，%)** | | | | |
| **Severe condition** | 22（59.46） | 10（43.48） | 12（85.71） | 0.011 |
| **Non-severe condition** | 15（40.54） | 13（56.52） | 2（14.29） | |
| **Underlying Conditions/(n，%)** | | | | |
| **Hypertension** | 24（64.86） | 13（56.52） | 11（78.57） | 0.314 |
| **Type 2 Diabetes** | 14（37.84） | 10（43.48） | 4（28.57） | 0.365 |
| **Coronary Heart Disease** | 25（67.57） | 16（69.57） | 9（64.29） | 1.000 |
| **Cerebrovascular Disease** | 14（37.84） | 10（43.48） | 4（28.57） | 0.365 |
| **Smoking History/(n，%)** | 6（16.22） | 3（13.04） | 3（21.43） | 0.833 |
| **Alcohol History /(n，%)** | 3（8.11） | 2（8.70） | 1（7.14） | 1.000 |
| **Time from Symptoms to Admission/[d，$\overline{X}$ ± s]** | 9.51±4.26 | 9.43±4.64 | 9.64±3.71 | 0.888 |
| **Length of Hospital Stay/[d，$\overline{X}$ ± s]** | 14.14±8.39 | 13.70±8.21 | 14.86±8.95 | 0.689 |
| **Hospitalization Cost/[10,000 RMB，Md(IQR)]** | 3.30±3.13 | 2.61±2.25 | 4.44±4.04 | 0.085 |
| **ICU Admission/[d，Md(IQR)]** | 0（0） | 0（0） | 0（0） | NA |
| **ICU Length of Stay/[d，Md(IQR)]** | 0（0） | 0（0） | 0（0） | NA |
| **BMI（kg·m$^{-2}$，$\overline{X}$ ± s）** | 23.61±3.12 | 23.74±3.50 | 23.25±1.93 | 0.685 |
| **Treatment/(n，%)** | | | | |
| **High-Flow Nasal Oxygen** | 20（54.05） | 8（34.78） | 12（85.71） | 0.003 |
| **Invasive Mechanical Ventilation** | 3（8.11） | 0（0） | 3（21.43） | 0.021 |
| **Non-Invasive Mechanical Ventilation** | 1（2.70） | 0（0） | 1（7.14） | 1.000 |
| **Complications/(n，%)** | | | | |
| **ARDS** | 11（29.73） | 3（13.04） | 8（57.14） | 0.013 |
| **Respiratory failure** | 11（29.73） | 3（13.04） | 8（57.14） | 0.013 |
| **Bacterial Pneumonia** | 3（8.11） | 2（8.70） | 1（7.14） | 1.000 |
| **Fungal Pneumonia** | 0（0） | 1（4.35） | 0（0） | 1.000 |
| **Cardiac Injury** | 1（2.70） | 1（4.35） | 0（0） | 1.000 |
| **Liver Injury** | 6（16.21） | 2（8.70） | 4（28.57） | 0.258 |
| **Kidney Injury** | 2（5.41） | 0（0） | 2（14.29） | 0.137 |
| **Lower Limb Thrombosis** | 4（10.81） | 2（8.70） | 2（14.29） | 1.000 |
| **Cerebral Infarction** | 3（8.11） | 3（13.04） | 0（0） | 0.430 |
| **Myocardial Infarction** | 0（0） | 0（0） | 0（0） | NA |
| **Prognosis /(n，%)** | | | | |
| **Survival** | 34（91.89） | 23（100） | 11（78.57） | 0.047 |
| **Death** | 3（8.11） | 0（0） | 3（21.43） | |

NA：Not Available

Table S2. Markers to identify VS with AUC>0.70, related to Figure 4.

| No. | Category | Name | AUC | *P* | FC |
|---|---|---|---|---|---|
| 1 | Protein | CO8G | 0.835 | 0.001 | 0.074 |
| 2 | Protein | LUM | 0.823 | 0.001 | 0.088 |
| 3 | Protein | CO4B | 0.820 | 0.002 | 0.134 |
| 4 | Protein | RTN4 | 0.804 | 0.007 | -0.869 |
| 5 | Protein | KNG1 | 0.795 | 0.009 | 0.115 |
| 6 | Protein | CFAD | 0.776 | 0.010 | 0.241 |
| 7 | Protein | CFAI | 0.776 | 0.022 | -0.036 |
| 8 | Protein | VTNC | 0.776 | 0.013 | 0.058 |
| 9 | Protein | CO8B | 0.773 | 0.012 | -0.087 |
| 10 | Protein | CFAB | 0.767 | 0.004 | 0.234 |
| 11 | Protein | CO8A | 0.767 | 0.007 | -0.024 |
| 12 | Protein | MMP9 | 0.767 | 0.008 | -1.558 |
| 13 | Protein | PI42A | 0.767 | 0.003 | -3.867 |
| 14 | Protein | EPCR | 0.764 | 0.007 | 0.568 |
| 15 | Protein | ANT3 | 0.761 | 0.007 | 0.087 |
| 16 | Protein | CO6 | 0.755 | 0.010 | 0.083 |
| 17 | Protein | GIT1 | 0.755 | 0.046 | 1.278 |
| 18 | Protein | LCAT | 0.755 | 0.029 | -0.046 |
| 19 | Protein | ATPB | 0.748 | 0.014 | -0.614 |
| 20 | Protein | CO4A | 0.748 | 0.014 | 0.239 |
| 21 | Protein | FHR5 | 0.748 | 0.029 | -0.791 |
| 22 | Protein | GRP1 | 0.745 | 0.009 | -3.593 |
| 23 | Protein | FIBA | 0.736 | 0.012 | -1.141 |
| 24 | Protein | FIBG | 0.736 | 0.015 | -1.198 |
| 25 | Protein | AMPN | 0.730 | 0.016 | 0.382 |
| 26 | Protein | APOH | 0.727 | 0.014 | 0.274 |
| 27 | Metabolite | Glucosamine-6-phosphate | 0.727 | 0.022 | -1.420 |
| 28 | Protein | GELS | 0.724 | 0.012 | 0.213 |
| 29 | Protein | SRC8 | 0.724 | 0.021 | -0.933 |
| 30 | Protein | GPIX | 0.720 | 0.015 | -1.185 |
| 31 | Protein | ERN1 | 0.717 | 0.075 | -1.931 |
| 32 | Protein | FIBB | 0.717 | 0.024 | -1.050 |
| 33 | Protein | NCAM1 | 0.717 | 0.011 | 0.072 |
| 34 | Protein | TYB4 | 0.714 | 0.032 | 0.818 |
| 35 | Protein | ICAM1 | 0.711 | 0.021 | -0.016 |
| 36 | Metabolite | Taurocholic acid | 0.708 | 0.023 | -2.181 |
| 37 | Metabolite | Glutamine | 0.708 | 0.050 | 0.187 |
| 38 | Protein | CD14 | 0.702 | 0.016 | -0.177 |
| 39 | Protein | TBB1 | 0.702 | 0.045 | -0.845 |
